# Selections and Abstracts

**Published:** 1902-05

**Authors:** 


### SELECTIONS AND ABSTRACTS.

### The Pelvic Floor and Its Injuries.*

Before reading my brief essay, I wish to be allowed the privilege of thanking the society most heartily for the invitation to read a paper before it. I consider I have been highly honored, and I hope my humble effort will not be altogether disappointing.

The subject to which I wish to call your attention this evening is not a new one by any means. It is a subject which involves very largely the health and happiness of the most important part of the human race—the women. I refer to injuries sustained by the pelvic floor during parturition.

In the first place, what is the normal pelvic floor and what is its function?

For practical purposes it is safe to say that the human species is a composite, the result of evolutionary processes. The requirements of higher life have necessarily modified anatomical structures to meet the new demands thrown upon them. The pelvic floor of the human female is a marked example of this. The upright position has made necessary the entire reconstruction of the plane of muscles closing the outlet of the pelvis. This plane of muscles and fascia contains clefts and the exit of the alimentary and genito-urinary canals. In those animals which habitually assume the horizontal position, all the muscular mechanism that is necessary, is to prevent the escape of the contents of these canals, except at voluntary intervals. To accomplish this certain circular muscles are thrown around the respective termini, and are called sphincters. The support of the abdominal viscera is maintained by the ventral abdominal wall. In the human subject it becomes necessary to specially modify the muscular mechanism of the pelvic outlet, so that, in addition to the sphincters, muscles of support

*Read by invitation before the St. Louis County Medical Society at Duluth, March 13, 1902, by William E Ground, M. D., member of the Western Medical and Gynecological Association, Etc., West Superior, Wis.

must be supplied. We therefore, then, have in the highly differentiated pelvic floor of the humnn female two distinct muscular layers, functionally and morphologically different. Those muscles having to do with sphincteric action are developed from the primitive sphincter cloaca, while those forming the pelvic diaphragm and originally the muscles of the caudal appendage are developed from the primitive flexors and adductors of the caudal vertebræ. Those muscles (the lavator ani) constituting the pelvic floor, or diaphragm proper, have been evolved originally from the flexors and extensors of the caudal end of the vertebral column. In the tailed animals it is the business of these muscles to move that appendage. When the upright position was assumed and tails were no longer required, these muscular structures were utilized to furnish support for the superimposed pelvic and abdominal viscera. Hence the modern pelvic floor.

In considering the myology of the pelvic diaphragm only those muscles that are actively concerned will be considered in detail. The superficial muscles, the sphincter ani, constrictor vaginæ, and transversus perinei, are only mentioned to be excluded from our discussion. The pelvic diaphragm is formed by a thin muscular sheet called as a whole the levator ani. It is composed of four paired muscles, the ischiococcygeus, the iliococcygeus, the pubococcygeus, and the puborectalis. This series of muscles take their origin from around the brim of the true pelvis, and are inserted into the sacrum, coccyx, and median tendinous raphe. The ischiococcygeus arises from the ischium, and is inserted into the lateral border of the lower part of the sacrum, and the upper part of the coccyx. The iliococcygeus takes its origin from the iliac portion of the obturator fascia, and is inserted into the lateral border of the lower part of the coccyx and median raphe. While these two paired muscles form a part of the sides of the pelvic floor, they cannot be said to form an important constituent element. To the pubococcygeus and the puborectalis is assigned this important function. The pubococcygeus arises from the back of the body of the pubic bone along an oblique line which extends from the lowest limit of the symphysis outward to the obturator canal, and to a limited extent, from the obturator facsia. From this origin the fibers pass back by the urethra, vagina, and rectum, and are inserted by a tendinous expansion upon the ventral surface of the lower part of the sacrum and coccyx. The puborectalis, or sphincter recti of Holl, "arises from the back of the lowest part of the symphysis under cover of the pubococcygeus, from the upper layer of the triangular ligament and from the pubes immediately below the symphysis. From this origin the fibers pass around the lower rectum, meeting with fibers from the opposite side to form a strong loop or girdle which slings the rectum and vagina up under the pubic bone." I wish to call particular attention to the fact that the fibers of this muscle pass not only around the rectum, but between the vagina and rectum. This is the muscle that receives the major part of the injury sustained during delivery. The process of evolution has radically changed this muscle, even more than its asssociates. While its influence upon the caudal vertebræ has diminished, its influence upon the rectum and vagina has increased. So much so has this been the case that Holl considers the puborectalis as the best developed of the muscles of the pelvic floor, whereas in the lower animals it is hardly discernible.

This muscle keeps the rectum and vagina pulled forward under the pubic bone, so that the anus occupies a position on a line from one tuber ischii to the other, and about half way between the tip of the coccyx and the symphysis pubis. This is the position when the puborectalis is intact, but when it is lascerated or has lost its tone, it allows the perineal body to recede; and the perineal flexure of the rectum and vagina is lost. The puborectalis forms a complete sling around the rectum and vagina, and by direct continuation of its fibers produces a sharp angulation of these canals about an inch from the skin surface.

The function of this muscular sheet is that of support, pure and simple. Take a young, healthy and well developed female—one that has grown as nature intended that she should, who has not been stultified by the evil influence of the modern fashion-plate—and we shall see the curves of beauty, as well as the utility of the human form divine. With the subject standing erect, we notice a marked curving forward of the vertebral column in the lumbar region.. This tilts the pelvis so that the plane of the pelvic brim is almost perpendicular, the promontory of the sacrum being almost directly above the lower border of the symphysis. This allows the weight of the abdominal contents to strike on the anterior abdominal wall and the pubic bone. The intra-abdominal pressure is then deflected almost directly backward and downward, impinging against the coccyx and the pelvic floor. It will readily be seen that the force exerted by the downward pressure of the abdominal and pelvic viscera is so deflected in each instance as to pass almost at right angles with the center of gavity, so that there is no direct pressure from above on the normal pelvic floor. On the other hand, destroy the anterior lumbar curve or impair the integrity of the pelvic floor, and the entire mechanism is changed. The plane of the pelvic inlet assumes a position more nearly horizontal, allowing the intra-abdominal forces to be exerted more nearly parallel to the line of gravity, *i. e.*, directly upon the pelvic floor; or if the pelvic floor be injured, the force falls directly upon the vaginal orifice.

It is practically impossible, as things now exist, for a woman to be delivered at full term without more or less injury to the pelvic floor. These injuries do not take place, as a rule, where they are plainly visible. The amount of damage done the pelvic floor in no way corresponds to the amount of lasceration of the vaginal mucous membrane or perineum proper. When we remember that the outlet of the birth canal, with a diameter of one to one and a half inches, is suddenly dilated until a ring of 10 to 12 inches in circumference is produced, it is not hard to understand why injury is inflicted.

The consequences of lacerations of the pelvic floor depends entirely upon the structures injured. If confined to the skin and external muscles, so far as support is concerned, little harm will follow, but if the deeper tissues, including the levator ani muscle, are involved, we find loss of perineal flexure of the rectum and vagina, with recession of the vagina and anus from the pubic arch, and relaxation of the pudendal aperture, with prolapse of the vaginal walls, due to diminished contractile power. The immediate danger occasioned by tears of the skin and mucous membrane are chiefly those of infection, unless the rectal sphincters are included in the traumatism, when incontinence of feces and intestinal gases will be added.

The remote pathology of pelvic trauma depends upon the extent of injury received by the levator ani muscle and its fascia. Lacerations of that part of the levator ani, known as the puborectalis, may take place at any point along its course, and its condition may be ascertained by passing the finger along over it as it passes by the sides of the vagina. Note the amount of resistance offered when pressure is made on this muscle laterally and behind. I have observed many cases where the tonicity of the levator ani was so destroyed that little resistance was encountered until the ligaments and pelvic bones were reached. The lacerations may occur immediately under a tear in the vaginal mucous membrane, or at another point, or the musculature of the pelvic floor may be extensively lacerated, and the mucosa and skin remain intact. As a result we have interference with proper involution of the vagina, vaginal prolapse, derangement of function of defecation and micturition, malposition of the uterus and appendages, followed by dynamic disturbance of the influences of intra-abdominal pressure, supervention of reflex phenomena, and a state of general ill health instituted, due to visceral ptosis.

It has lately become the general practice of obstetricians to make some attempt at repairing rents found, immediately after delivery. This practice, while beneficial so far as preventing avenues for infection and perhaps adding to cosmetic effects is concerned, is not calculated to restore the integrity of the pelvic diaphragm. Such suturing is almost invariably limited to the skin or superficial structures. Very rarely is a careful search made for deep and damaging lacerations. The tissues are at this time so swollen, edematous, and distorted that it is very difficult to properly place the sutures. Certainly repair work attempted on a bed, without assistants, retractors and a good light, is little more than a farce, so far as restoring functions is concerned. At best such operating simply restores the symmetry of the pudendal aperature, unless, however, the sphincter ani is torn, then immediate repair is imperative to prevent fecal incontinence.

On the whole, I believe better results will be obtained if superficial rents are sutured, and the general attempt at restoration left until involution has been fairly complete, say in the fourth week. Women should then be examined with a view to ascertaining the extent of trauma to which the pelvic floor has been subjected. If the anus has receded, the angulation is obliterated or greatly diminished, the vulva gaping so that straining brings into view everted vaginal mucous membrane—such a patient should be subjected to a thorough colpoperineorrhaphy before she has been much on her feet.

With regard to methods of operating, I shall speak only in a general way, giving nothing especially new, but rather gathering together a few facts from many sources, and making a method of procedure that appears to me to be a step in the right direction.

Those plastiques having to do only with the skin and mucous membrane, as before stated, are absolutely worthless against the ill effects of perineal relaxation and pelvic herniation. Most of the operations practiced—such as Emmet, Tait, etc.—do little more than restore the symmetry of the pudendal aperature, because they do not take into consideration the direct union of the puborectalis muscle and fascia, without suture en masse. They simply take a tuck in the vaginal mucous membrane, which, for the time being, contracts the introitus. While it is true that in many of these operations denudation is extended well up into the sides of the vagina, they are weak, in that they leave in the center an intervening patch of undenuded mucous membrane, and suture up along the sulci, or the denudation is not extensive enough, or the sutures are inserted in too superficial a manner or from the skin surface of the perineum, and do not, without intervening tissue, bring together the two lateral halves of the pelvic floor.

Referring to the anatomical diagrams again, we see how the puborectalis passes along beside and behind the vagina, and how it swings the vagina and rectum forward, so that when traumatism occurs the integrity of this muscle is destroyed, and pelvic relaxation results. These points are undisputed anatomical facts. This being the case, it seems to me the proposition to get down to the conditions actually at fault should admit of no dispute. Now there are only two operations proposed that actually deal with first principles, namely, the Harris operation, and the technique suggested and carried out by Goldspohn.

Harris in his operation, as you will remember, proposes to deal directly with the puborectalis at the point of laceration. An incision is made in the lateral walls of the vagina, and the puborectalis exposed, the point of laceration sought for, found, excised and sutured. The same may be done with the muscle on the other side. This of course shortens the muscle and pulls the recto-vaginal structures forward under the pubis. He repairs lacerations of the perineum in the usual way. I have had little personal experience with this operation, but did I not have a method that yielded satisfactory results, I should give the Harris operation an extensive trial, because I believe it to be founded on right principles.

The operation I have been executing during the last three or four years is in principle that described by Goldspohn in 1897. Dr. Goldspohn splits the rectovaginal septum well up toward the cervix, raises the vaginal flap, and with buried continuous catgut sutures the levator ani and its fascia together between the rectum and vagina, disregarding the point where rupture occurred. This is practically what I have been doing, except that I excise the vaginal flap, and suture with interrupted silkworm-gut sutures, in addition to bringing the puborectalis and fascia together with catgut. My reason for excising the flap and using interrupted silkworm-gut sutures instead of buried catgut, is the greater certainty of a more perfect apposition of the two sides, and the security with which I can keep the parts in that position until good union has taken place.

The operation is simplicity itself, there being no complicated suturing, such as is described in most text-books. The study of the wonderful patterns that have been cut out on the female perineum is certainly amusing, if not instructive. How I have wrestled with the difficult problems of perineal patchwork, and the gyrations of this or that man's suture in the past, no one knows but myself.

I begin operating by making three points with a clip of the scissors. One on each labium at the posterior limit of the remnant of the hymen, and the other in the posterior vaginal wall well up over any vaginal prolapse that may exist, usually about 2½ to 3 inches from the plane of the hymen. I denude between these points and at least half way up on the sides of the vagina in the region of the puborectalis. I would like to say right here that the bleeding is usually very free, and many points require ligating with catgut, as neglect to do this, and to depend upon the regular suturing to control hemorrhage, nearly proved fatal in a case of mine last fall. As it was I had to remove all of my sutures, and catch up the bleeding vessel. The woman was then so weak that I had no time to re-introduce the sutures, as the important matter then in hand was the saving of her life, which was accomplished by free hypodermoclysis. In about a week I curetted the raw surfaces, and replaced sutures the same as before, and never got a better result in my life.

I begin suturing at the upper angle of the wound, placing silk-worm-gut sutures deep and from the inside of the vagina, until the external border of the denudation is reached. Before tying these sutures I find and suture together the lateral halves of the pubo-rectalis with fine formalized pyoctanin catgut. I begin then from above, and tie the silkworm-gut sutures. Occasionally if there is considerable tension on the catgut produced by bringing the pubo-rectalis together, I tie the silkworm-gut down to the point of cat-gut suture, before finally securing this suture. I then proceed to tie the remainder of the silkworm sutures. We then have only a slight wound exposed at the skin surface, which can be readily closed with a few superficial sutures.

This operation has given me admirable results, even in the worst forms of pelvic floor relaxation and aggravated prolapse of the rectovaginal wall. I have been very successful with old cases of procidentia.

The points are to denude freely, secure puborectalis, and suture from within the vagina, for suture on the skin surface of the perineum is of no avail in restoring integrity of the pelvic floor.—*Northwestern Lancet.*

### A Brief Description of the Hospitals of Manila, with a Few Notes on Plague.*

The subject of plague may be somewhat inappropriate for this occasion, but my only excuse for offering it is that it is rather out of the regular run of diseases which we are wont to discuss, and it may be interesting to those who have not been brought in contact with, nor had any opportunity to study, this disease.

I will first briefly describe the hospitals of Manila and vicinity to give you a general idea of the manner our sick and wounded are looked after in our insular possessions across the seas.

The most important and largest hospital on the islands is the First Reserve, which was formerly the Spanish Military Hospital, situated in the Ermita district. This has accommodations for about 1,200 patients. The ward and administration offices consist of a conglomeration of structures of all sizes and shapes, composed of stone covered with stucco with metal roofs. These buildings are all of one story, only about four feet from the ground, and the hospital necessarily covers a great deal of ground space. The wards, as a general rule, are large and airy and well lighted. The operating-room is modern in every respect. In connection with this hospital is the clinical, pathological and bacteriological laboratory, which was under the direction of Dr. Strong, who, with his assistants, has done commendable work in the investigation of tropical diseases. The hospital has a refrigerating plant, which is situated on the banks of the Pasig river.

The Second Reserve, or Hospital Malate, in the Malate district, has about 250 to 300 beds. The building was transformed from a convent and is well suited for a hospital, with its high ceilings, wide corridors and polished hardwood floors. The operating-room is only a makeshift, and there is not the opportunity of doing much surgical work here. When I left Manila about six months ago, there was some talk of abandoning this building on account of the high rental, and because the requirements for the sick were diminishing.

---

*Read before the semi-annual meeting of the Southwestern Minnesota Medical Society, Pipestone, Minn., February 6, 1902. By Harry Morell, M. D., late Acting Assistant Surgeon United States Army, Litchfield, Minn.

The Third Reserve, or Hospital No. 3, has about 300 beds and is for the most part used as a hospital for the convalescent and chronic cases. It is here that the hospital corps men of the army are sent for instruction.

The Santa Mesa Hospital, or, as it is sometimes called, the "Nipa" Hospital, from the fact that the whole hospital is composed of nipa palm and bamboo. In connection with this hospital Dr. Ewing has his laboratory. He is a very enthusiastic bacteriologist, and has done some original work in the study of beri-beri. This hospital is arranged on the cottage system and has between 250 to 300 beds.

The convalescent hospital is situated on Corregidor island across the bay.

These complete the military hospitals of Manila, with the exception of the various regimental hospitals situated in and around the suburbs.

The quarantine station is across the bay at Marie Vales under the jurisdiction of the Marine hospital service, of which Dr. J. C. Perry is the chief officer, and who deserves great credit and praise for the manner in which quarantine work has been carried out.

The civil hospitals consist of San Juan de Dios, situated in the walled city, and has about 300 beds, used mostly by the native element.

On a lovely morning last May I took advantage of an opportunity to visit the civil hospitals in the outskirts of Manila.

The San Lazaro—this is the leper hospital—contained about 75 lepers at the time of my visit. A large part of the building in a separate wing is devoted to the treatment of prostitutes, native and Japanese. A large proportion of these present themselves regularly for treatment, while others are confined here and held until they are cured. The latter class comes mostly from the District of Sampaloc.

The most interesting sight was the Japanese ward, which is large, well lighted and scrupulously clean. It contained about 30 beds, and in this ward the Japanese women were confined. The view looking into this ward was very strange and oriental. All the women wore highly colored and gay kimonas, giving them a very grotesque appearance. They were lying around in easy attitudes, distinctively of the East.

The next place visited was the plague pest hospital. This was really a camp, as the patients were kept in tents. Here were seen some cases of the bubonic and pneumonic forms of plague. The patients were all natives. The mortuary contained the body of a native dead with plague and another which had just died of tubercular leprosy. There is a small crematory in connection with the hospital.

The Chinese hospital was next visited. It is supported, I believe, by the Chinese merchants of Manila, and is in charge of Chinese physicians. I cannot say it was in a good condition from a sanitary point of view. Here were many cases of Dhobie itch, leprosy, tuberculosis, beri-beri, tropical ulcer and calentura, a malignant form of malarial fever. The wards in this hospital are dimly lighted and odorous.

I next called at the Chinese pest house, which is in the same vicinity and contains plague cases only. The building is about 150 feet long and composed of bamboo framework covered with nipa, which is a species of palm. The floor consists of split bamboo. Bamboo cots covered with matting were arranged on both sides. The Chinese doctor who was in charge was very courteous and took a great deal of time in showing me the various cases of plague. At the time of my visit there were about fifteen or twenty cases. The patients were nearly all affected with the bubonic type of the disease, which is the most frequent form, especially during epidemics. One of the patients had maniacal delirium, which is sometimes associated with the acute stage of the disease. This patient had his arms fastened behind his back and then tied to the floor with Manila hemp. On inquiry as to why he was restrained so forcibly, I was informed he was "mucho loco" (very crazy).

On examination of the different cases, one will be struck with the variance of the symptoms and lesions. in some the prostration and vomiting is extreme with no glandular enlargement. In others the glandular enlargement and edema are well marked and very extensive. In these cases the buboes are very tender, and in some of the cases the pain was so very acute that the Chinese doctor had applied some solid extract of opium to the painful glands. This treatment seemed to give great relief, but had the disadvantage of being very dirty. In some of the cases where the glands had broken down and suppurated, the mixture of pus and opium emitted a very evil odor and was nasty to look upon. In India the ice bag is used, which is superior to this method in relieving the painful parts and has the advantage of being cleanly. It is in these forms where the bubo goes on to suppuration that the ganglionic merges with the septicemic, and the symptoms are those of a high grade of septicemia with stupor, delirium and more or less profound unconsciousness.

The disease has been classified into three types—(*a*) bubonic, (*b*) septicemic, (*c*) pneumonic.

In the bubonic form, which is the most common, the femoral glands are the most frequently affected. This is accounted for by the fact that the natives go barefoot. A short time ago some newspaper came out in large headlines with the information that a Chicago physician predicted that the dread scourge plague would sweep the continent of America from end to end. This ominous foreboding is possibly an exaggeration, but it cannot be denied that plague is slowly showing itself and making advance toward this continent. It is a fact that the cases have not been many and the ports visited by this disease have been few, but that is just the reason why every precaution should be taken by the health authorities to prevent cases from slipping in unawares. This is especially true at our seaport towns. In nearly all parts of the world, with the exception of North America, epidemics have occurred. At different times plague has shown itself. To-day commerce, trade and communication with the Orient has increased and multiplied, and it is in this that the danger lies. The international disturbances in China, the American war in the Philippines, the touching of transports at ports in China, Japan and Honolulu, the return of large numbers of troops from these countries, would have a tendency to propagate the disease.

The health and quarantine officials of the different governments are fully alive to the danger ahead, and are doing their utmost to effectually stamp out, or at least limit the pestilence. The Japanese government is, in this regard, second to none. A United States army transport inspection at Nagasaki by the Japanese authorities was witnessed by the writer. There were about 1,300 soldiers aboard, and the men were individually examined. Each health inspector was provided with a microscope, and any one who showed the least signs of the illness was gone over carefully, thoroughly examined, and a microscopic examination was made of his blood. Our own authorities at Manilla are extremely careful in this respect also. Before a transport clears the port for the United States all baggage is fumigated and an inspection tag applied to each piece. The ship's company is inspected and examined. The writer, with an assistant, was assigned to this duty on one of the transports clearing for the United States. The different crews were paraded under their chiefs, each individual was stripped, and the femoral glands were gone over carefelly, and any enlargements or tenderness sought for. The extremely good sanitary condition of Manila in regard to plague is, in a large measure, due to the very careful work done by the Marine hospital service in the Philippines.

The epidemic of plague at Tokio and Kobe was effectually stamped out by very active measures by the Japanese authorities. In Honolulu within the past two years over two millions of dollars have been appropriated to fight plague, and whole blocks of buildings have been destroyed, as in San Francisco, to combat this malignant malady. There have been more scourges of plague in the world than of any other epidemic disease.—*St. Paul Med. Journal.*

---

### CONSTIPATION—ITS TREATMENT WITHOUT DRUGS.

The theme is threadbare. This is because the condition is so common. Half the people one meets, nay, two-thirds of them, are victims. It clouds their lives, sours their dispositions, dims their mental and moral perceptions, casts a "bilious" shadow over their social and even their religious natures, poisons their secretions and renders their lives scarcely half worth living. It gives its subjects a disagreeable breath and a dirty complexion.

The train of evils following in its wake is almost endless. It is a constant menace through autoinfection and throws down the bars to every form of infection from without. It is the godfather of nearly all the chronic forms of disease, of liver and kidney failure, of diabetes and dyspepsia, of neurasthenia, nephritis and neuralgia. Headache, heart trouble and hysterics, with scores of others of the commonest and most incorrigible maladies that afflict humanity, often have their origin and are always aggravated by constipation.

What are its causes?

These, too, are numberless.

Diets that are too much concentrated.

Too much sugar, condiments, pastry.

Too little fruit—fresh, juicy sub-acid fruit—and too few watery and succulent vegetables.

Too rapid eating, with too little mastication.

Nervous abstraction while eating.

Too much drinking at the time of eating.

Too much strong coffee and tea.

Sedentary occupations. Too much indoor life and too little exercise.

Want of system and regularity in the matter of evacuations.

Abuse and over-use of cathartic medicines.

Imperfect breathing habits.

The list might be almost indefinitely extended.

What are the rational means of cure?

First, *correct all the bad habits.* Nothing can take the place of this injunction.

A concentrated diet is one that contains too little bulk and too much nutriment, or at least too much of some one or more of the proximate principles of food. It may be too much fat, too much starch, too much sugar or too much flesh. It is an unbalanced ration. Correct such a diet by eating less flesh, less rich puddings, gravies and dressings, and substituting fruits and vegetables.

Over-cooking often impairs an otherwise good dietary. This, however, does not apply to the cooking of flesh or the baking of bread. Despite the current fallacies as to raw meats, all flesh should be well cooked, but not burned. Bread also is improved by being well baked, or even twice baked, as this chemically changes much of the starch and aids digestion.

As to the manner of eating, the "quick lunch" mania prevails.

If we had the stomachs of the carnivora—the lynx, the leopard or the lion, or the gizzards of the hen hawk, turkey buzzard or eagle—we might tear fish and flesh into strips and swallow it whole.

But we haven't.

*Take time for every meal or don't eat it.*

No danger of starving.

Tanner fasted more than forty days, and a ten days' fast once or twice a year would be worth a small fortune to a majority of the average feeders.

Most people wash down their food with slops—tea, coffee, chocolate, milk, beer or wine. In time the salivary glands become atrophied for want of use. Masticate every mouthful long and well, after which you can swallow it without a drink to hurry it along.

Coffee and tea are responsible for a host of ills, and of these constipation is not one of the least.

The greatest harm comes to those who are confined indoors, compelled to live in ill-ventilated rooms and deprived of all health-giving exercise.

Get out of doors.

Open up windows.

Run races and ride bicycles.

Do something to make your blood circulate and to force you to breathe. Very few people breathe efficiently. With a lung capacity of nearly a gallon most people inhale less than a quart of air at each inspiration. It is no wonder that consumption has become so common as to be called the "Great White Plague." Rather it is a wonder that more of these collapsed lungs do not forget to do their duty and go into a decline.

There are mechanical aids. These are daily massage, following the line of the colon; but it must be both vigorous and persistent, not merely occasional and passive. When constipation has become a habit, nothing short of eternal vigilance and the constant cultivation and exercise of will-power will permanently overcome it. The use of the thousand and one nostrums is only palliative. They, in the end, confirm and perpetuate the evil they are aimed at.

Bending the body at the middle backward and forward, sidewise, twisting, gyrating, stooping, swinging and thrusting the arms upward, backward, forward, round and round, reaching, striking, pulling and pushing—all these motions are of value. Rapid walking, horseback riding—if the horse is not too easy in his gait—kicking, swinging the legs, squatting and rising rapidly many times repeated. Any motions or exercises that act upon the abdominal muscles, that stimulate the diaphragm, accelerate the breathing function and favor the peristaltic movement of the bowels, will aid in banishing the demons and hobgoblins that dance and devastate in the wake of this national, if not cosmopolitan, malady—*constipation.*—*The Dietetic and Hygienic Gazette.*

---

### MEDICAL AND SURGICAL PROGRESS.*

*Members and Guests of the Academy:*

I am deeply sensible of the honor and responsibility you have conferred upon me in selecting me to preside over the deliberations of this body during the year just past. The society, though young, not yet out of its swaddling clothes, is healthy and strong, and has added during the year 33 per cent. to its bodily strength, and bids fair to add luster to the fame already achieved in the broad field of medicine and surgery. Well may the members of the Academy of Medicine feel proud of the scientific work accomplished, and should stimulate us to renewed efforts. Nothing speaks better for the intelligence and progressiveness of the physicians of a community than a large membership and punctilious attendance upon their local medical organizations. None of us are so blessed with medical knowledge and experience as to not gain something by association and interchange of ideas with other medical men. The experience and insight of every one may be helpful to some one else; we, therefore, owe not only to ourselves, but to others, active

*Address of the retiring President of the Academy of Medicine of Los Angeles. Delivered January 10, 1902, by B. F. Church, M.D., Los Angeles, Cal.

cooperation in society work. To add diversion and relaxation, I would suggest an occasional social function or entertainment to partly relieve the constant strain placed upon us, which would also encourage a larger attendance at meetings.

### SCIENTIFIC PROGRESS.

In calling your attention to the year's progress in medicine and surgery, which is usual in addresses of this character, I shall not attempt to enumerate all of the advances of the year, but touch briefly upon a few of the more important.

### ANESTHESIA BY LUMBAR PUNCTURE.

The production of general anesthesia by sub-arachnoidal injection of cocain has been widely discussed during the past year or more, and clinically adopted by some of the leading surgeons of the country. Testimony as to its usefulness, up to the present time, is of the most conflicting nature. The procedure originated with Dr. Leonard Corning of New York, who practiced it in laboratory experiments as long ago as 1885. Tuffer has recently brought out a most favorable report of 400 cases. He refutes the reported mortality made by others, of 6 in 2,000 cases, and claims that in no instance could death be properly attributed to the injection. He is also just as positive that there is no danger of permanent nervous affections following the operation. Reclus, of France, however, is a vigorous and logical opponent of the method. From statistics he places a discouraging aspect upon the experiments so far conducted.

Our own writers seem to agree in preferring general anesthesia by chloroform or ether, except where, for any reason, these are contra-indicated and a substitute must be found. Governed, therefore, by these restrictions, the use of this method to produce general anesthesia would be greatly limited.

It is pretty well established that the present concensus of opinion is, that anesthesia by lumbar puncture is not applicable to children or extremely sensitive adults, particularly women, or where major pelvic or abdominal operations are required.

Granting the accomplishment of entire absence of physical suffering by use of the method, one can easily imagine the profound mental and physical shock which patients must undergo when witnessing major operations upon their own viscera.

This method of anesthesia does not relax the muscular system, a desideratum which weighs heavily against its bid for preference over ether and chloroform in the performance of difficult operations.

PARAFFIN INJECTIONS.

Prosthesis by means of paraffin marks an experimental research of great promise, and one deserving of widespread and painstaking trial.

According to Gersung, paraffin with a melting point of about 37 deg. centigrade, may be injected in tissue subcutaneously or otherwise, for the purpose of building up the parts. The paraffin becomes encapsulated with fibrin threads deposited through it, produces no irritation and is absorbed very slowly, if at all. He reports two cases of external nasal deformities, saddle bridge nose, being corrected by subcutaneous injection of paraffin. One of his cases after a lapse of two years showed no change in the results first obtained by the procedure.

Gersung also reports its successful use in a case of incontinence of urine due to traumatism. The paraffin was injected in the tissues around the neck of the bladder, the rigid paraffin impregnated tissues acting as a valve with desired results.

Haban has recently employed this agent in four cases of cystocele, with satisfactory results, by making the injections between the walls of the vagina and bladder, then inserting a pessary for twenty-four hours to permit the mass to harden in the proper position. The most practical and universal use of the method will, in all probability, be for correcting external nasal deformities.

PROTOZOON OF CANCER.

Dr. Gaylord, of Buffalo, makes rather a sweeping statement in a recent report that he had isolated the protozoic parasite of cancer. Few investigators, at present, however, are prepared to accept his deductions in toto. Max Schueller, of Berlin, made a preliminary report last year describing certain organisms, probably of an animal nature, which he, by original methods, had succeeded in cultivating from human carcinoma and sarcoma. He does not claim that the parasites occur freely in the blood of carcinomatous or sarcomatous patients as Dr. Gaylord does.

An editorial in the June number of the *Journal* of the American Medical Association has the following to say upon the subject: "It is clearly the duty of physicians and surgeons to not allow long established doctrines, such as the purely local nature of carcinoma in its early stages and its possible permanent curability at the time, to be overthrown or modified in the slightest by premature and unsupported statements of sincere but over-zealous investigators into the etiology of cancer. Great harm would result were the impression to grow that cancer is a blood disease sure to break out somewhere else if removed."

### OVARIAN TRANSPLANTATION.

Of interest, especially to the Gynecologists, are the experiments, made more particularly upon the lower animals, which prove that after complete extirpation of the ovaries auto or heterografting can be performed. The ovary in its new position functionating as a normal organ.

The honor of the original idea is conceded to Knauer, of Germany, whose first publication appeared in 1896. The object of Knauer was to ascertain if in animals ovaries extirpated and then transplanted in any other portion of the peritoneal cavity were capable of living and performing their function. His experiments consisted in removing, under strict antiseptic precautions, the two ovaries of rabbits and then grafting them in some other part of the peritoneal cavity. The results were such as to warrant the author in affirming that in the rabbit ovaries could be transplanted in any region of the peritoneal cavity and they would not only live but normally functionate. Experiments of Gregorieff confirmed the conclusions of the original investigator, who, out of twelve rabbits submitted to ovarian grafting, four were successfully fecundated.

Dr. R. T. Morris, of New York, who, by the way, deserves a division of the honor with Knauer for priority of these investigations, has gone still further, having extirpated the diseased ovaries of a patient, took a healthy portion of one of the glands and grafted it in the vicinity of one of the tubes. One month after leaving the hospital the woman became pregnant.

MASTOID DISEASE.

Recent collective experience demonstrates that where streptococci are found in the discharges of acute and middle ear disease, 80 per cent. of them will sooner or later, require radical surgical procedure. The discharge, therefore, of every acute case of otitismedia, with mastoid involvement, should be examined microscopically for the presence of pyogenic organisms to better enable us to arrive at a conclusion regarding mastoid operation.

In performing the mastoid operation the tendency of to-day is more and more toward radical work; the removal of every vestige of necrosed tissue or detritis, boldly following it to its farmost limit, lead where it may. Usually the Stacke-Schuartz operation is performed for the relief of chronic mastoiditis, the Schuartz operation in acute otitis-media with mastoid involvement and the Stacke in chronic middle ear and antrum disease where the cells of the mastoid are not involved.

SUBCONJUNCTIVAL INJECTIONS OF SALT SOLUTION.

The injection of weak, common salt solution under the bulbar conjunctiva as a local remedial agent was first brought before the profession by Prof. Wellinger in 1895. He first employed the method in treating ulcers of the cornea. The injections have since been employed, with marked success in opacities of the vitreous and some other intraocular affections. The fluid, if used not stronger than two per cent., produces no irritation, is quickly absorbed, and has a powerful depurating effect upon some conditions of cloudiness in the vitreous.

In conclusion, I would urge renewed interest and energy in medical society work. Let each member attend meetings at all times when possible, and take part in the proceedings, if nothing more than to enter into discussion upon the subjects in hand. Your experience will add to the general fund of information.

I would suggest also, that more attention be given to the clinical feature of the meetings. Practical experience, demonstrated by the presentation of patients before the members of the society is always of interest and value.—*Southern California Practitioner.*

### MALARIA.

The chief interest in connection with malaria at present centers in the relation of this disease to the anopheles mosquito. Mauser, for example, allowed himself to be bitten by mosquitoes fed in Rome on a case of tertian malarial fever. The period of inoculation was between ten and sixteen days; he then developed fever, and the parasites were found in the blood. Nine months later, when he had been apparently cured by quinine, he spontaneously developed malaria.

Young stated in a discussion before the British Medical Association, that in Hong Kong the occurrence of malaria was always associated with the appearance of the anopheles mosquitoes; and Manson urged that the natural conditions antagonistic to this insect should be discovered and employed to exterminate them. Fernside stated that it was impossible to eradicate the mosquito in India, because to do so would destroy the rice-swamps. Natal stated that the anopheles is attracted by dark colors and repelled by light colors, a hint that may be of practical use. On the other hand, Egbert states that in the mountains of Honduras, at an altitude far above the mosquito belt, malaria fever of both the quotidian and tertian types is freqent, and he thinks it is propagated by fleas.

The prophylactic treatment, of course, consists in avoiding mosquito bites, for which Bras employs the punkah or the electric fan, and Macgregor recommends extermination by filling the swamps or covering the pools with kerosene. He warns that results should not be expected for two or three years, and in the meantime prophylactic doses of quinine should be employed. Ewing contributes a valuable study of the life history of the malarial parasite which serves to show how incomplete is our knowledge of this organism at present. The relation of quinine to malarial hemoglobinuria is still unsettled.

Klein reports fifteen cases in which this complication followed almost immediately the administration of quinine. He cautions against the careless administration of the drug in large doses, and thinks that black water fever is the result of poisoning by it.

Smith and Taylor report two cases, one in an officer who had been taking quinine in moderate doses for a long time; the other in a negro soldier. The parasites appear to have been exceedingly few in the blood.

Two extraordinary complications are noted. The first, observed by Mellroy, was a negro who went into a state of coma lasting seven hours; bullæ developed upon the knees, and four weeks later they became gangrenous, The estivo autumnal parasites were found.

The second, observed by Ewing, was a girl who was brought to the hospital suffering from fever, vomiting and constipation. There was considerable albumin in the urine, and a diagnosis of typhoid fever with acute nephritis was made. At the autopsy the renal tubules were found packed with malarial parasites. Ewing concludes as the result of a study of recorded cases, that malaria may produce acute degeneration of toxic origin in the kidneys or focal necroses with numerous hemorrhages, and the parasites may be massed in the renal capillaries, causing hemorrhage and thrombosis. The last form occurs only in estivo autumnal infections.—*Philadelphia Medical Journal.*

---

### THE CONTAGIOUSNESS OF PSORIASIS.

Psoriasis is one of the most common of skin diseases, but one the origin of which is still obscure. The question is frequently asked by patients, "Is it catching?" and the invariable answer is, "No." But are dermatologists justified in making such a dogmatic statement? In 1889 a French physician, Destot, scarified his arm and inoculated it with a psoriasis scab. In a few days an eruption appeared, having all the characteristics of psoriasis, and this eruption occurred four times in two years. The diagnosis was probably correct, but, as in all similar cases, there is always the possibility of coincidence.

A similar case has been recently reported in which a man is supposed to have acquired psoriasis through the operation of tattooing, he not having suffered from the disease previously. On the whole, we can not regard the evidence in favor of the contagiousness of psoriasis as conclusive, one of the chief arguments against this view being the fact that it is very common for one married person to have the disease for years without passing it on to the other.—*Medical Press and Circular.*

---

### REPORT OF A CASE OF SUBCUTANEOUS INJECTION OF PARAFFIN.

My attention was attracted to this subject some time ago by a request from a young gentleman to alter the shape of his nose. He had a typical saddle nose, the result of a blow received some ten years ago.

After reading an account of the results obtained by Gersuny in these cases by the subcutaneous injection of soft paraffin, I determined to try it. The operation is very simple, and requires little time. In my case the patient left the operating room and went immediately to his work.

The usual preparation of the field of operation is, however, very necessary, as is also the thorough sterilization of all instruments used. After the injection of cocain, the paraffin, which has been previously boiled and allowed to cool, is drawn into the syringe and injected just under the skin until the depression is filled. It produces no irritation, and heals in place with little or no inflammation. In the course of two or three months it grows harder, and becomes of cartilaginous consistence.

The term paraffin used in this connection is misleading, as one naturally infers it is the hard paraffin which is used—the substance from which candles are made; it is soft paraffin, or "white vaseline," that should be used, which is a soft salve at the temperature of the room, and which emerges like a worm from the point of the needle.—*J. F. Lynch, in Virginia Medical Semi-Monthly.*

---

The Cow Pea is the title of the latest publication issued by the Experiment Farm of the North Carolina State Horticultural Society at Southern Pines, N. C. This book, neatly bound and illustrated, in plain and concise manner discusses the value and uses of this important crop, the cow pea. Every reader can get a copy free by writing to the Superintendent of Experiment Farm, Southern Pines, N. C.

